# Constipation—Its Treatment without Drugs

**Published:** 1902-10

**Authors:** 


### Constipation—Its Treatment Without Drugs.

First, *correct all the bad habits.* Nothing can take the place of this injunction. * * * Take time for every meal or don't eat it. * * *

Bending the body at the middle backward and forward, sidewise, twisting, gyrating, stooping, swinging and thrusting the arms upward, backward, forward, round and round, reaching, striking, pulling and pushing—all these motions are of value. Rapid walking, horseback riding—if the horse is not too easy in gait!—kicking, swinging the legs, squatting and rising rapidly many times repeated. Any motions or exercises that act upon the abdominal muscles, that stimulate the diaphragm, accelerate the breathing function and favor the peristaltic movement of the bowels will aid in banishing the demons and hobgoblins that dance and devastate in the wake of this national if not cosmopolitan malady, constipation.—*The Dietetic and Hygienic Gazette.*

